# Hypertriglyceridemia‐induced acute necrotizing pancreatitis: Poor clinical outcomes requiring revisiting management modalities

**DOI:** 10.1002/jgh3.13061

**Published:** 2024-04-13

**Authors:** Yazan Abboud, Meet Shah, Benjamin Simmons, Kranthi Mandava, John E M Morales, Fouad Jaber, Saqer Alsakarneh, Mohamed Ismail, Kaveh Hajifathalian

**Affiliations:** ^1^ Department of Internal Medicine Rutgers New Jersey Medical School Newark New Jersey USA; ^2^ Department of Internal Medicine University of Missouri‐Kansas City Kansas City Missouri USA; ^3^ Division of Gastroenterology and Hepatology Rutgers New Jersey Medical School Newark New Jersey USA

**Keywords:** acute pancreatitis, hypertriglyceridemia, necrotizing pancreatitis, plasmapheresis

## Abstract

Hypertriglyceridemia‐induced acute pancreatitis (HTG‐AP) is the third most common cause of AP after gallstones and alcohol. Supportive measures, intravenous insulin, and plasmapheresis are possible treatment modalities for HTG‐AP; however, definitive guidelines evaluating the best therapeutic approach are not clearly established. We present a rare case of a 42‐year‐old male without known comorbidities who was found to have HTG‐AP. Despite early initiation of intravenous insulin and plasmapheresis and the initial decline in his triglycerides level, his condition was complicated by necrotizing pancreatitis and subsequent multi‐organ failure. Future studies are warranted to evaluate the role of plasmapheresis in HTG‐AP and its efficacy.

## Graphical abstract

While supportive measures, intravenous insulin, and plasmapheresis are possible treatment modalities for hypertriglyceridemia‐induced acute pancreatitis (HGT‐AP), definitive guidelines evaluating the best therapeutic approach are not clearly established. We report a 42‐year‐old male who was found to have HGT‐AP and developed necrotizing pancreatitis and subsequent multi‐organ failure despite early initiation of intravenous insulin and plasmapheresis. Future studies are warranted to assess risk factors associated with outcomes of HTG‐AP, and to evaluate the role of plasmapheresis in HTG‐AP and its efficacy.

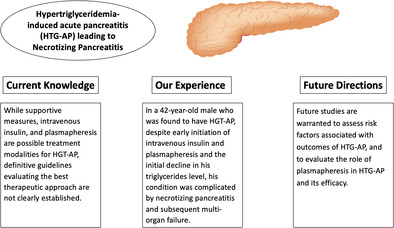

## Introduction

Hypertriglyceridemia is the third most common cause of acute pancreatitis after gallstones and alcohol, accounting for 10% of all cases. Acute necrotizing, defined when >30% of the pancreatic gland is necrotic, occurs in 5–10% of all acute pancreatitis cases and is associated with high morbidity and mortality.^1^ Management of acute pancreatitis includes supportive measures such as aggressive fluid resuscitation and pain management, along with etiology‐specific measures. For instance, the treatment of hypertriglyceridemia‐induced acute pancreatitis (HTG‐AP) includes dietary fat restriction, intravenous insulin, and potentially plasmapheresis, depending on the severity of disease.^2^ Having said that, there are limited data evaluating outcomes between different management modalities and no definitive guidelines on the best management approach for HTG‐AP. Furthermore, there is a paucity of the literature on HTG‐AP complicated by necrotizing pancreatitis and its management. We report a rare case of HTG‐AP complicated by necrotizing pancreatitis, despite the early initiation of intravenous insulin and plasmapheresis.

## Case report

**Figure 1:**
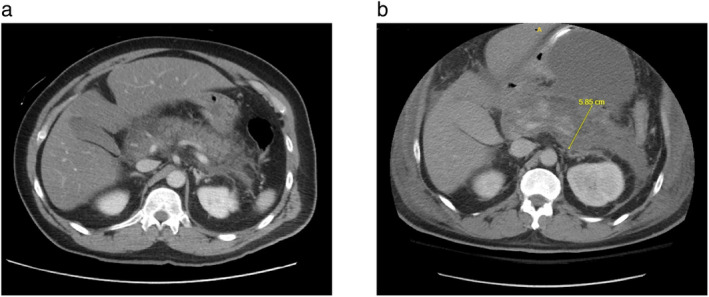
(a) Computed tomography of the abdomen and pelvis with intravenous contrast consistent with acute interstitial pancreatitis with severe hepatic steatosis and hepatomegaly. (b) Computed tomography of the abdomen and pelvis with intravenous contrast consistent with necrotizing pancreatitis with peripancreatic fluid collection, partially loculated fluid within the left anterior pararenal space, increased acute necrotic collection exerting increased mass effect on the adjacent stomach, increasing loculation and peripheral enhancement of the collection, and interval increase in the amount of intra‐abdominal ascites, specifically in the perihepatic space.

A 42‐year‐old male with no known medical history presented with severe epigastric abdominal pain radiating to the back, accompanied by nausea and non‐bloody non‐bilious vomiting for a few hours. Upon examination, the patient was tachycardic (HR 118) and tachypneic (RR 34). Laboratories were significant for leukocytosis (WBCs 14.9), hyponatremia (Na 128), lactic acidosis (LA 3.3), elevated lipase (1850), and hypertriglyceridemia (Triglycerides 3350 mg/dL). Computed tomography (CT) of the abdomen and pelvis was consistent with acute interstitial pancreatitis (Fig. 1). The patient denied any history of alcohol use or any medications or supplements intake. He was started on intravenous fluids and was admitted to the ICU and began receiving continuous intravenous insulin 4 h after presentation. Fluids and insulin were then held and the patient underwent an emergent session of plasmapheresis 6 h after admission; he was given 2000 units of heparin along with plasmapheresis directly into the catheter site. Triglyceride levels down‐trended from 3350 to 774 after the plasmapheresis and subsequently decreased to 383. On day 17 of admission, the triglyceride levels recurred to 1131, with repeat CT suggestive of necrotizing pancreatitis (Fig. 1a and S1, Supporting information). His hospital course was complicated by acute respiratory distress syndrome and aspiration pneumonia, for which he was started on broad‐spectrum antibiotics and required intubation. He also developed acute renal failure requiring intermittent hemodialysis and abdominal compartment syndrome induced by necrotizing pancreatitis, which led to the patient receiving another session of plasmapheresis and CT‐guided peripancreatic fluid drain. Despite these measures, the patient's clinical condition deteriorated to the point of needing urgent exploratory laparotomy with necrosectomy due to his multi‐organ failure. The patient later passed away from a cardiopulmonary arrest attributed to multi‐organ failure.

## Discussion

We present a rare case of a 42‐year‐old male without any known comorbidities who had HTG‐AP, which was complicated by necrotizing pancreatitis and subsequent multi‐organ failure despite the early initiation of intravenous insulin and plasmapheresis and an initial decline in his triglycerides level.

Previous data reporting HTG‐AP complicated by necrotizing pancreatitis is extremely limited. A study performed by de Oliveira *et al*. suggested that a key mediator of necrosis and subsequent multi‐organ failure in AP was the increase in pancreatic triglyceride lipase (PNLIP) in adipose tissue; PNLIP would then generate nonesterified fatty acids (NEFA), which had previously been shown to cause multi‐organ failure even in the absence of triglycerides via endothelial cell damage, leading to vascular leak.^3^ However, even in the absence of NEFA, patients with triglyceride levels more than 1000 mg/dL are at increased risk of acute pancreatitis, with data suggesting triglycerides greater than 2000 mg/dL associated with severe disease.^4^ Despite the >1000 mg/dL triglyceride level being generally accepted as the threshold for precipitation of HTG‐AP, it may prove prudent to consider early initiation of therapy in patients with triglyceride levels between 500 and 1000 mg/dL to prevent complications with pancreatic necrosis. In a retrospective study including 224 patients with HTG‐AP, 122 of whom had triglyceride levels >1000 mg/dL (group 1) and 102 of whom had triglyceride levels between 500 and 1000 mg/dL (group 2), 10 individuals developed pancreatic necrosis in each group. Of those 20 participants, 2 died from complications related to necrotizing pancreatitis, including one from the 500–1000 mg/dL group.^5^ This raises the possibility that triglyceride levels >500 mg/dL may be a sufficient threshold to begin considering the risk of progression to pancreatic necrosis. Further studies assessing the risk of necrosis in HTG‐AP patients with triglyceride levels <1000 mg/dL are needed.

Existing literature evaluating the outcomes of HTG‐AP based on the timing of intravenous insulin and plasmapheresis initiation is scarce. Although insulin therapy has been used as a conservative measure in the treatment of HTG‐AP, studies have not shown any significant therapeutic benefit to intensive versus non‐intensive insulin therapy.^6^ Furthermore, a randomized study comparing insulin therapy to fasting did not show a more rapid decline in triglyceride levels with insulin therapy when compared with fasting, suggesting that insulin therapy is not superior to fasting. Therefore, we aimed to further cover plasmapheresis as an alternative therapy to treating HTG‐AP in our discussion.

A previous case series of two patients who had HTG‐AP complicated by necrotizing pancreatitis showed different outcomes based on the timing of initiating plasmapheresis.^7^ The first patient was a 36‐year‐old male with triglyceride of 6460 mg/dL who underwent plasmapheresis 20 days after symptoms onset and ended up passing away from multi‐organ failure. However, the other patient who was a 52‐year‐old male with triglyceride of 3540 mg/dL underwent plasmapheresis early and had normalization of triglyceride levels and resolution of his condition. Furthermore, another previously reported case of a 42‐year‐old man with HTG‐AP complicated by necrotizing pancreatitis with triglyceride of 11 800 mg/dL developed multiple organ failure; however, the patient received emergent plasmapheresis and intravenous insulin, which normalized his lipid abnormalities and successfully resolved his condition.^8^ These prior reports suggest the possible benefit of early initiation of plasmapheresis after symptoms onset, which can normalize triglyceride levels and improve outcomes. An observational study performed by Gubensek *et al*., however, showed that a delay in the initiation of plasmapheresis did not influence survival, with there being no difference in mortality in the early and late plasmapheresis groups.^9^ Although there was no benefit to survival with regard to the timing of plasmapheresis seen in this study, it was noted that patients treated with citrate anticoagulation had lower mortality rates compared with those treated with heparin. In our case, the patient was treated with heparin during his plasmapheresis sessions; unfractionated heparin has been shown to increase plasma levels of free fatty acids due to their enhancement of plasma lipolytic activity.^10^ Therefore, the choice of anticoagulation during plasmapheresis, in addition to the timing of plasmapheresis, is another avenue for further investigation to take place to determine the health outcomes of patients with HTG‐AP.

Despite the early initiation of intravenous insulin and plasmapheresis in our patient, his condition did not improve and was complicated by necrotizing pancreatitis with multi‐organ failure and subsequent death. It should be noted that pancreatitis itself can lead to abnormalities in lipid levels, leading the patient to go into the circle of acute pancreatitis and HTG‐AP.^11^ This may potentially explain what occurred in our patient, who had recurrent elevations of his triglycerides and required several sessions of plasmapheresis.

Recent 2023 guidelines by the Writing Committee of the American Society for Apheresis suggest that plasmapheresis has a potential role in severe HTG‐AP, with the strength of evidence being 1C, meaning that while it is a relatively strong recommendation, it is based on low‐quality evidence and may change when higher evidence is available.^12^ Overall, plasmapheresis is a recommended treatment option for HTG‐AP with worrisome features. A randomized clinical trial comparing plasmapheresis with conventional medical management in HTG‐AP showed that the plasmapheresis arm achieved a more rapid decline in triglycerides and a lower incidence of complications.^13^ Having said that, a recent prospective multicenter study involving 276 patients with HTG‐AP showed no difference in the incidence of organ failure, but did show higher ICU admissions in patients who received plasmapheresis compared with conventional medical management.^14^ Our patient had several worrisome features warranting emergent plasmapheresis; however, despite its early initiation and the initial decline in triglycerides, he developed necrotizing pancreatitis that did not respond to treatment and was eventually complicated by multi‐organ failure. Future studies are warranted to further evaluate the management of HTG‐AP and the best time to initiate plasmapheresis and assess its efficacy.

In conclusion, we present a rare case of HTG‐AP complicated by necrotizing pancreatitis despite early management with intravenous insulin and plasmapheresis. There is no firm evidence on the best management modality for HTG‐AP. While supportive management, intravenous insulin, and plasmapheresis are treatment options, there is a lack of sufficient data favoring one approach over the other. Plasmapheresis is a recommended management modality for HTG‐AP with severe features. Future studies are warranted to assess risk factors associated with outcomes of HTG‐AP, with the goal to improve outcomes and prevent sequelae.

## Supporting information


**Figure S1.** Change in triglyceride levels and significant clinical events during the hospital course.
