# Supplementary figures and images for: Hypertriglyceridemia‐induced acute necrotizing pancreatitis: Poor clinical outcomes requiring revisiting management modalities
Source: JGH Open. 2024 Apr 13;8(4):e13061. doi: 10.1002/jgh3.13061 (PMC11015164; doi:10.1002/jgh3.13061)

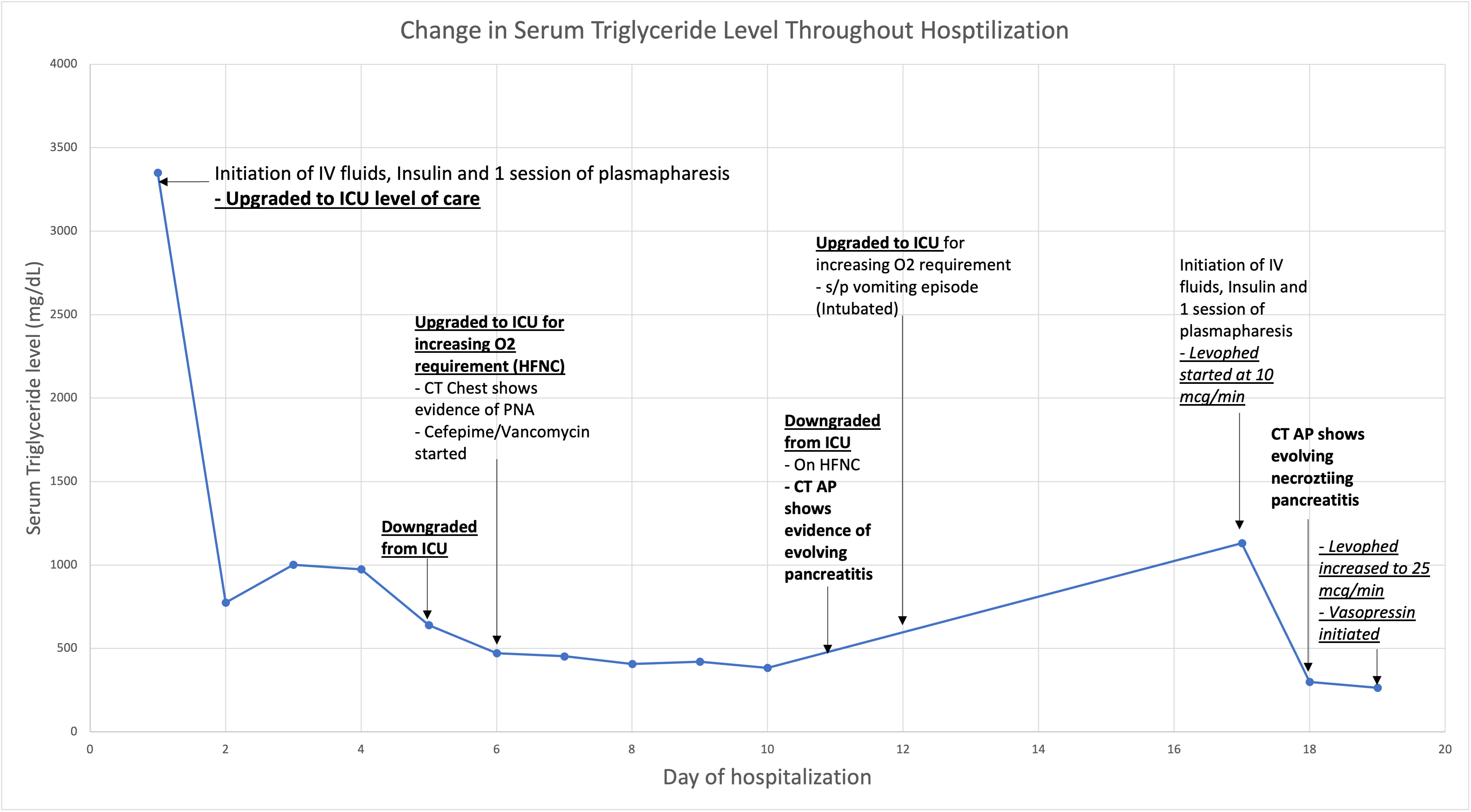

Supplement: Supplementary file 1 — Figure S1. Change in triglyceride levels and significant clinical events during the hospital course. [file JGH3-8-e13061-s001.png]
